# Weight Regain and Ingestive Behavior in Women after Metabolic Surgery

**DOI:** 10.3390/nu15173670

**Published:** 2023-08-22

**Authors:** Jessica G. Nicanor-Carreón, Neda Seyedsadjadi, Blair Rowitz, Marta Yanina Pepino

**Affiliations:** 1 Division of Nutritional Sciences, University of Illinois at Urbana-Champaign, Urbana, IL 61801, USA; jgn3@illinois.edu (J.G.N.-C.);; 2 Department of Food Science and Human Nutrition, University of Illinois at Urbana-Champaign, Urbana, IL 61801, USA; 3 Carle Illinois College of Medicine, Urbana, IL 61801, USA; 4 Department of Surgery, Carle Foundation Hospital, Urbana, IL 61801, USA

**Keywords:** bariatric surgery, sleeve gastrectomy, gastric bypass, sweet cravings, weight regain, ingestive behavior, eating behavior, sweetness, alcohol

## Abstract

This study investigated associations between maladaptive ingestive behaviors and weight regain in women who underwent metabolic surgery 2–10 years ago. Using a web-based survey, we assessed emotional, external, and restrained eating (Dutch Eating Behavior Questionnaire—DEBQ), food cravings (Food-Craving Inventory—FCI), and other behaviors (e.g., Eating Disorder Examination Questionnaire—EDE-Q; Alcohol Use Disorder Identification Test-Concise—AUDIT-C) in 36 women (42.9 ± 9.5 years old) post-surgery. We found that weight regain was specifically associated with increased frequency of cravings for sweets (r = 0.43), higher global scores in the EDE-Q (r = 0.38), and time elapsed since surgery (r = 0.35; all *p*’s < 0.04). Multiple regression analysis revealed that the association between weight regain and sweet cravings interacted with time after surgery (*p* = 0.04), with the strongest association observed in women assessed closer to the surgery (i.e., 2.0–2.8 years). The combination of time after surgery and its interaction with sweet cravings accounted for 31% of the individual variations in weight regain (*p* = 0.005). Notably, among participants who reported alcohol consumption (31 of 36), 55% had an AUDIT-C score indicating hazardous drinking. These findings highlight the relevance of attending to patients’ reports of frequent sweet cravings and screening for alcohol use to enhance strategies tailored to prevent weight regain and alcohol-related health problems post-surgery.

## 1. Introduction

Obesity represents a significant and pressing global health concern, characterized by an excessive accumulation of body fat that poses a substantial risk of developing various chronic diseases, including type 2 diabetes [T2D], cardiovascular disease, and metabolic syndrome, among other medical comorbidities [1]. Epidemiological data from the United States collected between 2017 and 2020 indicate a concerning prevalence rate of 42% for obesity (as reviewed in [2]). Weight loss surgery, or bariatric/metabolic surgery, is the most effective treatment for severe obesity and its concomitant comorbidities [3].

Currently, the most common metabolic surgical procedures performed in the United States, with similar trends worldwide, are sleeve gastrectomy (SG) (76.5%) and Roux-en-Y gastric bypass (RYGB) (21.2%) [4,5]. A systematic review and meta-analysis of published long-term outcomes have shown that RYGB and SG provide substantial and sustained weight loss, with patients achieving approximately 60% of excess weight loss [6]. Along with weight loss, there is a significant reduction or even remission of conditions such as T2D, hypertension, and dyslipidemia [3], which leads to an increase in life expectancy [7].

Despite the substantial weight loss achieved through metabolic surgeries, it is important to recognize that obesity is a chronic disease that requires long-term management even after the initial bariatric surgery [8]. After reaching the nadir weight (usually around two years post-surgery [9]), there is a risk of weight regain that is of primary concern as it may lead to the reappearance of chronic conditions and a subsequent decline in quality of life [10]. The significance of using the percentage of excess weight loss (% EWL) as an indicator for evaluating the success of surgery diminishes as the risk of weight regain increases after reaching the nadir weight. For instance, patients may still be classified as having achieved successful weight loss (typically defined as % EWL ≥ 50) even if they have regained a significant amount of the lost weight [10]. Therefore, it is important to consider measures of weight regain when assessing the long-term success of metabolic surgeries.

The prevalence of weight regain post-surgery varies widely in the literature, as there is no consensus on the most appropriate measure, cut-off point, or timing of assessment. However, the percentage of weight regain calculated from the maximum weight lost (% MWL), which considers the kilograms regained in relation to the initial weight loss, has demonstrated superior clinical significance compared to other measures of weight regain. A cut-off point of ≥20% MWL has been associated with worsening quality of life, decreased satisfaction with surgery, and the progression of diabetes, hyperlipidemia, and hypertension [9]. Identifying characteristics and behaviors linked to weight regain following metabolic surgeries holds promise for tailoring personalized strategies to sustain weight loss.

Previous studies in our laboratory have shown acute improvements in eating behavior following surgery [11,12,13]. These included a decrease in the pleasure elicited by sweet taste, a reduction in the frequency of food cravings (including cravings for sweets), a decreased influence of emotions and external food cues on eating behavior, and a decrease in the prevalence of food addiction. These positive behavioral changes were observed shortly after surgery, typically around 4–6 months, when participants followed dietary recommendations and achieved a weight loss of approximately 20% [11,12,13]. However, whether these positive outcomes persist long-term and are associated with weight resistance regain remains unknown.

Several research studies have identified factors associated with weight regain post-surgery; however, the findings are inconsistent. For example, a recent systematic review of the literature found mixed results when studies evaluated the correlation between weight regain after surgery and physical activity, picking and nibbling eating, macronutrient consumption, age, and gender, among others [10]. This is likely related to a wide variation in the study methods, such as different indexes of weight regain measures and cut-offs, timing of assessment, surgery type, and statistical analyses.

A recent study found positive associations between weight regain quantified as % MWL and various post-surgery behaviors, such as increased sedentary time, fast food intake, eating when feeling full, binge eating, losing control when eating, and frequently weighing [14]. Other studies have reported a positive association between weight regain and emotional eating [10,15,16] and increased sweet consumption [17] (as reviewed in [10]). In addition, specific characteristics have also been associated with a greater weight regain post-surgery, including being younger, having a longer time since the surgery, experiencing more depressive symptoms [14,18], and having problematic patterns of alcohol use [17].

Regarding postoperative alcohol use, there is a notable increase (almost double) in the risk of developing an alcohol use disorder following RYGB and SG procedures (as reviewed in [19]). One proposed mechanism contributing to this phenomenon is the alteration in alcohol pharmacokinetics due to surgery-induced changes in stomach anatomy. SG and RYGB result in faster and higher blood alcohol peaks when individuals consume the same amount of alcohol as before surgery [20]. Additionally, some researchers suggest the possibility of “addiction transfer”, where individuals may shift their addictive behaviors from food to alcohol after surgery [21].

This study aimed to assess the associations between maladaptive ingestive behaviors after surgery and the percentage of weight regain in women who underwent metabolic surgery 2–10 years ago.

## 2. Materials and Methods

### 2.1. Participants

For recruitment, we used a laboratory registry based on participants who had previously consented to being contacted for future research studies. We invited 111 women (21+ years old) who underwent SG or RYGB between 2 and 10 years ago to participate in an online study between June 2020 and November 2021. The recruitment was limited to women because the laboratory registry contact list primarily consisted of women, representing approximately 80% of patients undergoing metabolic surgery [22]. We excluded women with adjustable gastric banding (AGB), participants with metabolic surgery <2 or >10 years ago, or potential participants who reported being pregnant or breastfeeding during the study. Informed consent was obtained from all subjects involved in the study, which was approved by the University of Illinois at Urbana-Champaign Institutional Review Board (IRB #20841).

### 2.2. Study Procedures and Questionnaires

**Figure 1:**
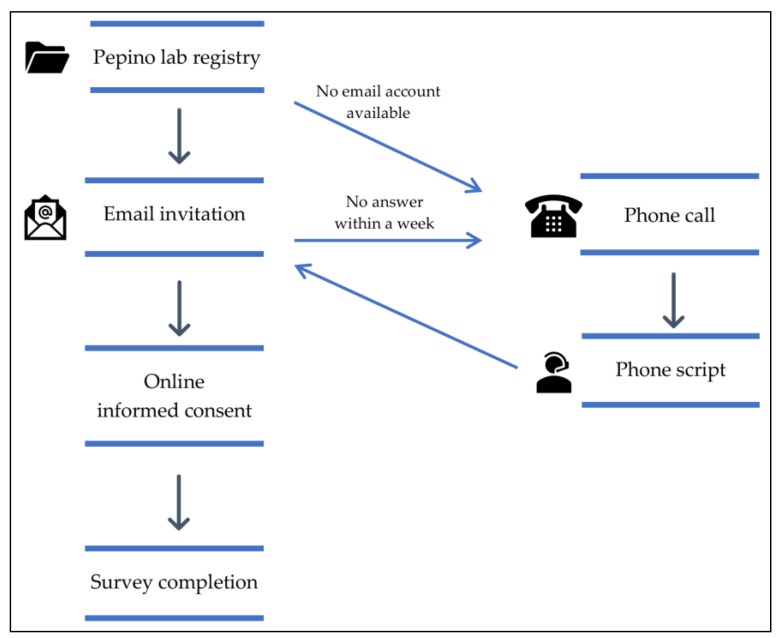
Study flow.

Due to the constraints imposed by the COVID-19 pandemic, in-person research was unfeasible, and therefore a self-administered web-based survey was designed for participants to complete from their homes. We emailed potential participants an online informed consent and a link to complete the web-based survey. We attempted to contact participants up to three times, including a phone call whenever the email address was unavailable (Figure 1). Data was collected using Qualtrics XM software (Qualtrics, Provo, UT, USA), and participants had the option to receive a USD 20 (Amazon gift card) as compensation for their time. The survey could be completed in 40–50 min, saved for continuing to answer later, and included the following questionnaires:

1. Sociodemographic questionnaire. It includes personal information such as age, year of birth, sex, race, ethnicity, height, weight before surgery, current weight, lowest (nadir) weight after surgery (and date), type of bariatric surgery (and date), and pregnancy or breastfeeding status;
2. Food-Craving Inventory (FCI) [23]. This validated tool measures the self-reported frequency of cravings for foods in general and specific foods. It encompasses four subscales: the frequency of cravings for high fats, carbohydrates, sweets, and fast-food fats in the past month, and it also obtains a total food craving score. Participants select their answers using a 5-point Likert scale (1 = never, 5 = always);
3. Dutch Eating Behavior Questionnaire (DEBQ) [24]. This questionnaire evaluates restrained, emotional, and external eating. Restrained eating refers to consciously restricting food intake to control body weight; emotional eating considers eating in response to negative feelings such as stress or loneliness; and external eating refers to eating in response to external food cues such as sight or smell. Participants select their answers using a 5-point Likert scale (1 = never, 5 = very often);
4. Eating Disorder Examination Questionnaire (EDE-Q) [25]. It assesses restraint, eating, shape, and weight concern over the past 28 days and obtains a global score that reflects the severity of eating disorder psychopathology. Participants select their answers using a 7-point Likert scale (0 = none of the time, 6 = every time);
  - Additional eating disorder questions. We added questions about bariatric surgery-related eating behavior, such as loss of control when eating, grazing food between meals, and subjective overeating based on questions on the EDE-Bariatric Surgery Version (EDE-BSV) interview [26];
5. Yale Food Addiction Scale (YFAS) version 2.0 [27]. This scale assesses addictive-like eating behavior in the past month according to the substance-related and addictive disorders section in the Diagnostic and Statistical Manual of Mental Disorders (5th ed.; DSM–5) [28]. The scale allows the classification of participants into different categories depending on their food addiction symptoms: mild, moderate, and severe. Participants select their answers using an 8-point Likert scale (0 = never, 7 = every day);
6. Alcohol Use Disorders Identification Test-Concise (AUDIT-C) [29]. This is a brief version (3 items) of the AUDIT questionnaire (10 items). Each item has five answer choices valued from 0 to 4 points. A score of 3 or more in women suggests hazardous drinking behavior or active alcohol use disorder.

The measure of weight regain chosen for this study was % MWL, as it has been previously shown to have better clinical significance than other measures [9]. We used self-reported data to calculate weight regain using the following formula:

Percentage of the maximum weight lost (% MWL): [100 × (current weight − nadir weight)]/(pre-surgery weight − nadir weight) [9].

### 2.3. Statistical Analyses

Statistical data analyses were performed with SPSS version 27 for Windows (IBM Corp, Armonk, NY, USA). We used the Kolmogorov–Smirnov and Shapiro–Wilk tests to evaluate the distributional properties of variables. To achieve a normal distribution, variables, including frequency of cravings for sweets and time after surgery, were transformed using base-10 log transformation, and the variable % MWL was transformed using a square root. However, the frequency of cravings for carbohydrates and eating concerns did not achieve normality distribution and were analyzed with non-parametric tests.

Correlations between variables were analyzed using Pearson’s or Spearman’s correlation coefficients, as appropriate. To ensure we could detect a moderate correlation (r = 0.45) in our study, we calculated the required sample size (N observations) using specific statistical parameters. We chose a two-sided test with a significance level of 5% (α = 0.05), which indicates the threshold for considering results statistically significant. Additionally, we aimed for a power of 80% (β = 0.2), which represents the probability of correctly rejecting the null hypothesis when it is false, i.e., the probability of detecting an actual correlation if it exists. After performing the calculations, we found that we would need 36 observations (*n* = 36) to detect moderate correlations and fewer for stronger ones to achieve the desired power and significance level.

Multiple linear regression analyses were conducted to analyze the association between weight regain as the dependent variable and the following predictors: frequency of cravings for sweets, time after surgery, global scores in the EDE-Q, the interaction term of global scores in the EDE-Q × time after surgery, and the interaction term of the frequency of cravings for sweets × time after surgery. The frequency of cravings for sweets, time after surgery, and EDE-Q scores were centered on their means before running the linear regression analyses to reduce collinearity among predictor variables. We tested collinearity with Variance of Inflation (VIF) in regression models, and VIF values less than three were considered non-collinear. Based on their time after surgery, women were assigned to three groups: first tertile, 2.0 to 2.8 years after surgery; second tertile, 2.9 to 4.4 years after surgery; and third tertile, 4.5 to 6.9 years after surgery.

## 3. Results

### 3.1. Participants’ Characteristics

Of the 111 women invited to participate in the study, 16 could not be reached because of wrong contact information, 13 were not interested in participating, 10 were interested but did not complete the survey, and 31 did not reply to our invitations. Forty-one women completed the online questionnaires; however, we excluded four individuals because their surgery was <2 or >10 years ago, and one underwent AGB. Thus, this study included 36 women. The characteristics and ingestive behavior-related outcomes of all participants are summarized in Table 1.

We found that 9 of 36 (25%) participants scored positively for food addiction (FA). Interestingly, as a group, those without and with FA reported having similar Body Mass Index (BMI) at the time of the study (without FA: 32.9 ± 1.4; with FA: 35.8 ± 2.5 kg/m^2^; *p* = 0.33) and before surgery (without FA: 48.5 ± 1.6; with FA: 49.2 ± 2.7 kg/m^2^; *p* = 0.82).

### 3.2. Weight Regain and Ingestive Behavior

Weight regain was positively associated with time elapsed since surgery (r = 0.35, *p* = 0.04), global scores in the EDE-Q (r = 0.38, *p* = 0.02), and frequency of cravings for sweets (r = 0.43, *p* = 0.01). However, weight regain did not correlate with the frequency of cravings for other foods (high fats: r = 0.30, *p* = 0.08; fast foods: r = 0.17, *p* = 0.32; carbohydrates: r = −0.14, *p* = 0.41); scores in the DEBQ: restrained eating (r = −0.02, *p* = 0.91); emotional eating (r = 0.26, *p* = 0.13); external eating (r = 0.29, *p* = 0.09); or women’s age (r = 0.22; *p* = 0.21).

Multiple regression analysis revealed that after controlling for the frequency of cravings for sweets and time elapsed since surgery, global scores in the EDE-Q were no longer associated with weight regain, and that time elapsed since surgery moderated the association between weight regain and the frequency of cravings for sweets (Table 2). The time elapsed since surgery and its interaction with the frequency of cravings for sweets accounted for 31% of the individual variations in weight regain (F_(5, 35)_ = 4.17; *p* = 0.005).

**Figure 2:**
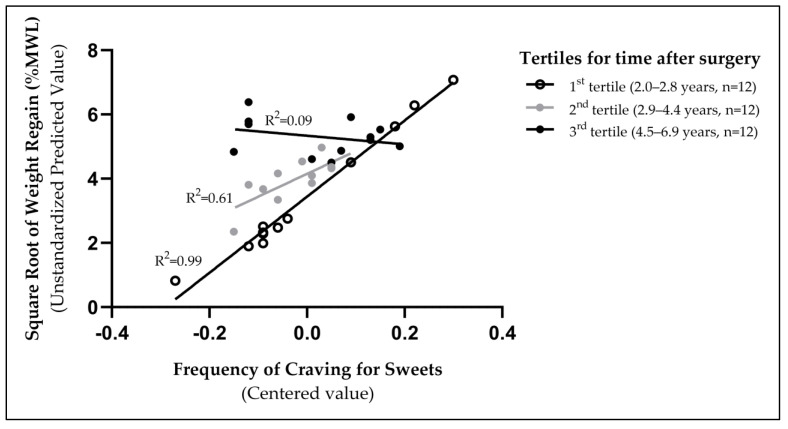
The association between the frequency of sweet cravings and weight regain was strongest for those assessed closer after surgery (1st tertile, 2.0–2.8 years).

To interpret the interaction between the frequency of cravings for sweets and the time elapsed since surgery as they related to weight regain, we grouped women based on their time after surgery using tertiles (Figure 2). As illustrated, the association between the frequency of sweet cravings and weight regain was strongest in the group closer to the surgery (1st tertile = 2.0–2.8 years; R^2^ = 0.99) and then weakened as time passed from surgery (2nd tertile = 2.9–4.4 years; R^2^ = 0.61, and 3rd tertile = 4.5–6.9 years; R^2^ = 0.09). The three groups of women categorized based on time elapsed since surgery were of similar age (*p* > 0.98) and BMI (*p* > 0.25).

We also explored participants’ characteristics and ingestive behavior-related outcomes, stratifying women into two groups of weight regain with a cut-off of 20% MWL (Table 3). Compared to women in the <20% MWL group, women in the ≥20% MWL group had increased global scores in the EDE-Q (*p* = 0.04). In particular, they scored higher in the shape (*p* = 0.05) and weight (*p* = 0.01) concern subscales and were more likely to have a food addiction diagnosis (*p* = 0.03) and lose control when eating (*p* = 0.05). Interestingly, there was a trend that women in the <20% MWL group were more likely to engage in hazardous drinking than women in the ≥20% MWL group (*p* = 0.06).

## 4. Discussion

The primary finding of this study is that weight regain was positively associated with the frequency of cravings for sweets and that this relationship was moderated by time elapsed since surgery. The strongest association was observed for women assessed closer to the surgery period (i.e., 2.0–2.8 years), typically when the highest rate of weight regain occurs after surgery [9]. The combination of time elapsed since surgery and the interaction of this variable with sweet cravings accounted for 31% of the individual variations in weight regain. Additionally, higher weight regain was associated with increased scores in eating disorder measures, food addiction, and loss of control when eating. Notably, 55% of the women who reported alcohol consumption had an AUDIT-C score indicative of hazardous drinking. These findings align with previously identified specific ingestive behaviors that can contribute to weight regain after metabolic surgery [10,14,17] and emphasize the need for close monitoring and addressing dietary habits as part of the medical nutrition therapy during postoperative follow-up visits.

Several studies have identified sweetness-related phenotypes as *pre*-surgery predictors of weight loss. For example, Perez-Leighton and collaborators found that having a strong desire for sweetness was associated with greater weight loss in the first year after RYGB but not after SG [30]. Similarly, Smith et al. found that higher preoperative ratings of liking for sweet mixtures with varying fat concentrations and more frequent preoperative food cravings were associated with greater weight loss six months after RYGB but not after SG [31,32]. The authors suggested that, in contrast to SG, RYGB anatomical and metabolic modifications could be more effective in correcting the altered reward circuits in people with obesity, at least shortly after surgery. For instance, participants pre-RYGB who had lower activation in the ventral tegmental area in response to high-sugar and fat mixtures and greater activation changes at 2 postoperative weeks lost more weight at 6 months [32]. However, Smith et al. showed that this correction of the neural processing of reward stimuli in the mesolimbic pathway is not long-lasting and depends on postoperative eating behaviors, and that these *pre*-surgery phenotypes were found to be poor predictors of weight loss maintenance and weight regain after surgery [31], highlighting the greater relevance of *post*-surgery indicators [14]. In fact, some findings suggest that the factors influencing initial weight loss and weight regain after surgical [17] or non-surgical interventions [33] are entirely different.

Previous research, including our own, has shown improved eating behavior shortly after surgery [11,12,13,31]. There is also evidence that these changes can impact the success of the surgery. For example, patients who experienced a greater reduction in cravings for sweets at 6 months (post-RYGB) and 1 year (post-SG) compared to pre-surgery tend to have better weight loss in the first year following surgery [31]. However, evidence suggests that maladaptive eating behaviors can recur or emerge long-term after surgery [34]. In line with this evidence, the reported frequency of cravings for sweets, scores in emotional eating, and prevalence of food addiction for the women in this study resemble more previous results obtained *pre*-surgery than those obtained 4–6 months *post*-surgery [12,13]. In accordance with this observation, Tsouristakis and collaborators found that by the fourth year following surgery, sweet cravings were no longer different from pre-surgery [35].

Interestingly, when we look at these phenotypes by weight regain groups (Table 3), we find that lower weight regain is associated with a decreased prevalence of food addiction and a trend for decreased sweet cravings. This suggests that the reduced frequency of cravings for sweets and remission of food addiction observed shortly after surgery [12,13] may persist long-term in some patients and potentially contribute to weight maintenance. Other studies have investigated the association between weight-control dietary practices and weight regain post-surgery. Notably, avoiding the consumption of “sweets” has been associated with less weight regain [17], but “cutting out sugar-sweetened beverages” has not [14]. These discrepancies likely arise from variations in the specific sweet-related variables measured and the methods used to define weight regain. Alternatively, it is worthwhile to consider that consuming non-sugary-sweetened beverages may also trigger cravings for “sweet”. In other words, replacing sugar-sweetened-beverages with non-sugary sweetened beverages may not help with weight maintenance.

Our study found that 44% of the women enrolled self-reported experiencing loss of control (LOC) when eating. This percentage significantly exceeds the reported figures for patients assessed four years after SG (11%) [36] and aligns closely with the percentage reported in patients pre-bariatric surgery (40%) [37]. This eating behavior is relevant as LOC is a component of eating disorders such as binge eating or bulimia nervosa [38], requiring intensive and specialized treatment [38]. LOC when eating has been associated with poorer weight loss and greater weight regain after surgery [14,39], consistent with our findings (Table 3).

Another form of eating pathology pertinent to patients who underwent bariatric surgery is picking, nibbling, or grazing on food. A previous study reported that 39% of participants engaged in grazing around four years after SG surgery [36]. However, we found a significantly higher prevalence. Nearly all of the participants in our study self-reported engaging in this behavior (89%), with 66% of them grazing on food at least once every week. Devlin et al. have shown that the pre-surgery and third-year prevalence of participants grazing at least once every week are similar [37]. Grazing after gastric bypass surgery has been positively correlated with weight regain [40], consistent with our findings that 100% of the participants in the ≥20% MWL group reported engaging in this behavior. Participants in this group also have significantly higher eating disorder scores than the <20% MWL group and above the threshold that has been previously used by Gero et al. [41] to define an “unhealthy” group (>2.5). This highlights the need to find ways to prevent and treat eating disorders post-surgery, especially in patients with considerable weight regain.

Noteworthy, when comparing the group with the lower weight regain to the group with the higher weight regain, the former had a lower prevalence of food addiction (10% vs. 44%) but a trend to have a higher prevalence of hazardous drinking (68% vs. 33%). This observation is consistent with findings from a recent study [42] and the concept that some patients may substitute one reinforcer, “food,” for another, namely “alcohol,” which has been commonly referred to as “addiction transfer.” Previous research on a prospective multicenter cohort sample using the AUDIT (10-item) revealed that the prevalence of alcohol use disorder following RYGB surgery escalated from 7% pre-surgery to 16% at a 7-year follow-up. Moreover, the cumulative incidence of alcohol use disorder symptoms at the 5-year mark was 21% [43]. Our study identified that among the participants who consumed alcohol, 55% exhibited an AUDIT-C (3-item) score indicative of hazardous drinking. These findings emphasize the necessity of increasing patients’ awareness regarding alcohol consumption after undergoing metabolic surgery and its potentially harmful consequences.

These study findings should be interpreted in light of several limitations. Firstly, our sample size was small, as only a limited number of participants completed the web-based survey at one time post-surgery. Additionally, the survey was conducted during the COVID-19 pandemic, which could have altered participants’ eating and drinking behaviors (as reviewed in [44,45,46]). However, it is important to note that our main finding regarding the association between sweet cravings and weight regain aligns with and extends previously published research conducted before the COVID-19 pandemic [10,17]. Secondly, anthropometric measurements were self-reported, introducing some potential errors. However, in the context of bariatric populations, self-reported weights have been shown to be reasonably accurate when compared to measured weights [47]. Thirdly, our study focused exclusively on ingestive behaviors related to weight regain and did not consider other factors, such as anatomical adaptations (e.g., pouch or sleeve dilation) or physical activity and sedentarism, which could also influence weight recurrence [10,48]. Nevertheless, one strength of studying ingestive behaviors is that they may be relatively easier to modify than anatomical or physiological factors.

Lastly, it is important to acknowledge that our findings are limited to a specific sample of middle-aged women, primarily of Caucasian ancestry, the majority of whom underwent SG. Therefore, the results cannot be generalized to men or the broader bariatric population. However, it is worth noting that SG is currently the most common metabolic surgery performed worldwide, and it is predominantly carried out on women.

## 5. Conclusions

These findings emphasize the relevance of addressing patients’ reports of frequent sweet cravings post-bariatric surgery and implementing strategies to regulate them. Approaches such as pharmacologic therapies and cognitive strategies [49,50] could potentially aid in preventing weight regain, particularly in the early stages post-surgery. Furthermore, in line with previous reports and likely exacerbated by the COVID-19 pandemic, our study highlights a high prevalence of hazardous drinking within this population. This observation underscores the importance of monitoring alcohol consumption in addition to nutrient intake after surgery to ensure comprehensive patient care and support.

## Figures and Tables

**Table 1:** Participants’ characteristics and ingestive behavior-related outcomes.

Participants’ Characteristics	All, N = 36
Type of surgery (*n* (%))	SG: 30 (83%)
	RYGB: 6 (17%)
Age (years)	42.9 ± 9.5
Race (*n* (%))	White/Caucasian: 30 (83%)
	Black/African American: 5 (14%)
	Other: 1 (3%)
Ethnicity (*n* (%))	Non-Hispanic: 33 (92%)
	Hispanic: 1 (3%)
	Other: 2 (5%)
Time after surgery (years) ^a^	3.3 ± 1.1
Pre-surgery Body Mass Index (BMI) (kg/m^2^) ^a^	47.3 ± 3.5
Current BMI (kg/m^2^)	33.6 ± 7.4
% Excess Weight Loss (EWL)	66.6 ± 25.4
Weight regain: % Maximum Weight Lost (MWL) ^a^	18.2 ± 13.6
Time after surgery to reach nadir weight (years/*n* = 35) ^a^	1.2 ± 0.6
**Ingestive behavior-related outcomes**	
**Food-Craving Inventory**	
High Fat	1.9 ± 0.5
Carbohydrates ^a^	1.7 ± 0.5
Sweets ^a^	2.3 ± 0.5
Fast Food	2.4 ± 0.7
Total Cravings	2.1 ± 0.4
**Dutch Eating Behavior Questionnaire**	
Restrained eating	3.1 ± 0.6
Emotional eating	2.6 ± 0.9
External eating	2.8 ± 0.6
**Eating Disorder Examination Questionnaire**	
Restraint	2.2 ± 1.3
Eating concern ^a^	0.9 ± 0.8
Shape concern	3.3 ± 1.5
Weight concern	2.7 ± 1.5
Global score	2.3 ± 1.1
**Yale Food Addiction Scale** (*n* (%))	
No food addiction	27 (75%)
Food addiction	9 (25%)
Mild	1 (11%)
Moderate	2 (22%)
Severe	6 (67%)
**Loss of control when eating** (*n* (%))	
No	20 (56%)
Yes	16 (44%)
At least 1/28 days (monthly)	5 (31%)
At least 4/28 days (weekly)	11 (69%)
**Grazing** (*n* (%))	
No	4 (11%)
Yes	32 (89%)
At least 1/28 days (monthly)	11 (34%)
At least 4/28 days (weekly)	21 (66%)
**Loss of control when grazing** (*n* (%))	
No	19 (53%)
Yes	17 (47%)
**Alcohol use** (*n* (%))	
No	5 (14%)
Yes	31 (86%)
Non-hazardous drinking	14 (45%)
Hazardous drinking	17 (55%)

Values are means and standard deviations, except for those variables not following a normal distribution indicated by ^a^, which are median and semi-quartile range values.

**Table 2:** Multiple linear regression analysis for the association between weight regain (dependent variable), cravings for sweets, EDE-Q global score, time after surgery, and interactions.

n	Adj. R^2^	Predictor	B Coefficient	Standard Error	T	*p* Value	VIF
36	0.31	Frequency of cravings for sweets	5.20	3.28	1.59	0.12	1.66
		EDE-Q global score	0.13	0.38	0.35	0.73	1.76
		Time after surgery	4.93	2.13	2.31	**0.03**	1.17
		Frequency of cravings for sweets × time after surgery	−44.74	21.15	−2.12	**0.04**	2.09
		EDE-Q global score × time after surgery	1.61	2.47	0.65	0.52	1.99

Values in bold indicate *p* < 0.05.

**Table 3:** Participants’ characteristics and ingestive behavior-related outcomes by the weight regain group.

Participants’ Characteristics	<20 % MWL (*n* = 20)	≥20 % MWL (*n* = 16)	*p* Value
Type of surgery (*n*(%))	SG: 16 (80%)	SG: 14 (88%)	0.45
	RYGB: 4 (20%)	RYGB: 2 (12%)	
Age (years)	40.8 ± 8.3	45.6 ± 10.5	0.13
Race (*n*(%))	White/Caucasian: 18 (90%)	White/Caucasian: 12 (75%)	0.47
	Black/African American: 2 (10%)	Black/African American: 3 (19%)	
	Other: 0 (0%)	Other: 1 (6%)	
Ethnicity (*n*(%))	Non-Hispanic: 18 (90%)	Non-Hispanic: 15 (94%)	1
	Hispanic: 1 (5%)	Hispanic: 0 (0%)	
	Other: 1 (5%)	Other: 1 (6%)	
Time after surgery (years) ^a^	3.0 ± 0.9	4.3 ± 1.2	0.09
Pre-surgery Body Mass Index (BMI) (kg/m^2^) ^a^	47.8 ± 5.2	47.3 ± 2.5	0.73
Current BMI (kg/m^2^)	30.6 ± 5.8	37.5 ± 7.6	**<0.01**
% Excess Weight Loss (EWL)	80.4 ± 18.5	49.4 ± 22.6	**<0.0001**
Weight regain: % Maximum Weight Lost (MWL) ^a^	7.3 ± 6.4	37.4 ± 11.9	**<0.0001**
Time after surgery to reach nadir weight (years/*n* = 35) ^a^	1.4 ± 0.7	1.2 ± 0.2	0.37
**Ingestive behavior-related outcomes**			
**Food-Craving Inventory**			
High Fat	1.8 ± 0.5	1.9 ± 0.4	0.55
Carbohydrates ^a^	1.7 ± 0.4	1.9 ± 0.6	0.99
Sweets ^a^	2.0 ± 0.3	2.8 ± 0.6	0.09
Fast Food	2.4 ± 0.9	2.5 ± 0.6	0.71
Total Cravings	2.1 ± 0.5	2.2 ± 0.4	0.28
**Dutch Eating Behavior Questionnaire**			
Restrained eating	3.1 ± 0.7	3.2 ± 0.6	0.74
Emotional eating	2.4 ± 0.8	2.7 ± 1.1	0.31
External eating	2.7 ± 0.6	3.0 ± 0.6	0.09
**Eating Disorder Examination Questionnaire**			
Restraint	2.1 ± 1.3	2.4 ± 1.3	0.58
Eating concern ^a^	0.4 ± 0.6	1.6 ± 0.8	0.09
Shape concern	2.8 ± 1.6	3.8 ± 1.2	**0.05**
Weight concern	2.1 ± 1.4	3.4 ± 1.4	**0.01**
Global score	2.0 ± 1.1	2.8 ± 1.0	**0.04**
**Yale Food Addiction Scale** (*n* (%))			
No food addiction	18 (90%)	9 (56%)	**0.03**
Food addiction	2 (10%)	7 (44%)	
Mild	0 (0%)	1 (14%)	1
Moderate	0 (0%)	2 (29%)	
Severe	2 (100%)	4 (57%)	
**Loss of control when eating** (*n* (%))			
No	14 (70%)	6 (38%)	**0.05**
Yes	6 (30%)	10 (63%)	
At least 1/28 days (monthly)	2 (33%)	3 (30%)	0.65
At least 4/28 days (weekly)	4 (67%)	7 (70%)	
**Grazing** (*n* (%))			
No	4 (20%)	0 (0%)	0.08
Yes	16 (80%)	16 (100%)	
At least 1/28 days (monthly)	7 (44%)	4 (25%)	0.23
At least 4/28 days (weekly)	9 (56%)	12 (75%)	
**Loss of control when grazing** (*n* (%))			
No	12 (60%)	7 (44%)	0.26
Yes	8 (40%)	9 (56%)	
**Alcohol use** (*n* (%))			
No	1 (5%)	4 (25%)	0.11
Yes	19 (95%)	12 (75%)	
Non-hazardous drinking	6 (32%)	8 (67%)	0.06
Hazardous drinking	13 (68%)	4 (33%)	

Values are means and standard deviations, except for those variables not following a normal distribution indicated by ^a^, which are median and semi-quartile range values. Values in bold indicate *p* ≤ 0.05, and those underlined indicate a trend (*p* ≤ 0.09).

## Data Availability

The data presented in this study are available upon request from the corresponding authors. The data are not publicly available in accordance with the consent provided by participants on the use of confidential data.
